# New magnetic resonance imaging findings in patients with polymyalgia rheumatica

**DOI:** 10.1590/0100-3984.2023.56.1e3-en

**Published:** 2023

**Authors:** Marcello H Nogueira-Barbosa

**Affiliations:** 1 Associate Professor of Radiology, Faculdade de Medicina de Ribeirão Preto da Universidade de São Paulo (FMRP-USP), Ribeirão Preto, SP, Brazil. Email: marcello@fmrp.usp.br.

A new study of patients with polymyalgia rheumatica (PMR), using magnetic resonance imaging (MRI) of the shoulders and hips, is published in the previous issue of **Radiologia Brasileira**^(1)^. Unlike other rheumatic diseases such as rheumatoid arthritis, spondyloarthritis, psoriatic arthritis and gout, PMR is a condition that is relatively unknown to most radiologists. However, various imaging techniques have been shown to help define the diagnosis^(2-4)^. For example, the European League Against Rheumatism/American College of Rheumatology criteria for diagnosing PMR include ultrasonography findings, which can be used in order to confirm the presence of subdeltoid bursitis, biceps tenosynovitis, glenohumeral synovitis, trochanteric bursitis, and hip synovitis^(4)^.

Although the diagnosis of PMR is based on scores determined from clinical and blood tests criteria, the usual clinical manifestations of the disease, in isolation, are nonspecific. In addition, there is no reference standard for the diagnosis of PMR; such difficulties make the diagnosis of the disease a challenge^(4)^. Various diagnostic imaging modalities have been employed to rule out differential diagnoses and comorbidities. Imaging evaluation can also be necessary to identify findings related to large vessel vasculitis and cranial arteritis, which may coexist with PMR^(4)^.

In some cases, radiographs can be utilized to rule out calcifications due to crystal deposition disease or erosive arthritis, findings that are generally not expected in PMR. Ultrasonography has been employed to identify synovitis, tenosynovitis, and bursitis, which typically have a symmetrical distribution and are seen in regions commonly affected by the disease^(2,4)^. Other imaging methods that have been used include positron emission tomography, which can be especially useful in cases that are not responding to the treatment of choice^(4)^.

In clinical practice, MRI has occasionally been used for the diagnosis of PMR, and some studies in the literature have shown the potential usefulness of the method for that purpose^(5-7)^; the images obtained can demonstrate manifestations of synovitis, tenosynovitis, and bursitis in the shoulders and hips, although those changes are also nonspecific. The study conducted by Leão et al.^(1)^ and published in **Radiologia Brasileira** confirms the high prevalence of subdeltoid bursitis and joint effusion on MRI examinations of the shoulders and hips of patients with PMR. Notably, it is also one of the first studies to identify a high prevalence of capsulitis and peritendinitis on MRI in patients with PMR. That finding opens the possibility that new diagnostic criteria can be incorporated, which could increase the relative importance of MRI in the study of PMR, given that it is more accurate than is ultrasonography for identifying capsular changes and peritendinous edema.

One major limitation of the study in question is that it did not evaluate a control group of asymptomatic volunteers or groups of patients with other diseases. Nevertheless, the results are promising and should encourage further research on the use of imaging techniques and, in particular, the applications of MRI, in patients with PMR. The article authored by Leão et al.^(1)^ merits special attention because it expands the body of knowledge regarding PMR.
